# Multiple Rod Construct for Occipito-Cervical Fusion: Overcoming Challenges With Enabling Technologies

**DOI:** 10.7759/cureus.107690

**Published:** 2026-04-25

**Authors:** Bharat R Dave, Arjit Vashishtha, Ajay Krishnan, Shivanand C Mayi, Ravi Ranjan Rai, Mirant B Dave, Mukesh Patel, Mikeson Panthackel, Amritesh Singh, Saurabh S Kulkarni, Yogenkumar Adodariya

**Affiliations:** 1 Spine Surgery, Stavya Spine Hospital and Research Institute, Ahmedabad, IND; 2 Spine Surgery, Bhavnagar Institute of Medical Sciences (BIMS), Bhavnagar, IND; 3 Neurosurgery, Stavya Spine Hospital and Research Institute, Ahmedabad, IND; 4 Orthopedics, University College of Medical Sciences (UCMS) and Guru Teg Bahadur (GTB) Hospital, Delhi, IND

**Keywords:** craniovertebral junction, enabling technologies, intra-operative ct and navigation guidance, multiple-rod construct, occipito-cervical fusion surgery

## Abstract

Introduction

Occipito-cervical fusion is a well-established procedure for numerous cranio-vertebral junction (CVJ) anomalies. Owing to the complex biomechanics at play in this region, rigid internal fixation with pedicle screws and rods is needed to provide stability for fusion and good clinical outcomes. However, the rates of implant failure are still worrisome, especially in individuals with compromised anatomy and physiology. We describe a method for using multiple rod constructs for instrumentation across the CVJ, making the construct biomechanically stable, achieving high fusion rates, and preventing complications such as implant failure.

Methodology

This is a retrospective observational study with 10 patients who underwent occipito-cervical fusion with navigation-guided cervical pedicle screw fixation and multiple rod constructs, with a minimum follow-up of 12 months. Clinical parameters, including Visual Analog Scale (VAS), Neck Disability Index (NDI), Nurick grade, and modified Japanese Orthopaedics Association (mJOA) score, were evaluated pre-operatively and at follow-up. Similarly, radiological parameters such as occiput to axis (O-C2) lordosis, subaxial cervical spine (C2-C7) lordosis, and clivus-canal angle (CCA) were evaluated. Assessment of fusion was done by CT scan at 12 months post-operatively.

Results

All patients improved in terms of clinical and radiological outcomes post-operatively. Early mobilization on post-op day 1 with a soft cervical collar was possible in all the patients. No evidence of implant failure was seen in any of the cases. On follow-up CT scans, bony trabeculae formation was seen in all the patients.

Conclusion

Multiple rod construct for stabilizing the CVJ for occipito-cervical fusion is associated with early mobilization and clinical as well as radiological improvement due to the biomechanically stable construct provided by multiple anchorage in the cervical spine and occiput. This leads to high fusion rates, especially in patients with compromised anatomy and physiology.

## Introduction

Occipito-cervical fusion (OCF) is a well-established procedure for numerous cranio-vertebral junction (CVJ) pathologies of congenital, inflammatory, traumatic, or infective etiologies [1,2]. Due to the inherent complex biomechanics of the region, rigid internal fixation is necessary to provide stability and ultimately achieve bony fusion. Historically, uninstrumented occipito-cervical fusion was performed with autologous structural bone grafts (iliac crest, fibula, or rib), which required prolonged halo-vest immobilization in the post-operative period [3,4]. Instrumentation around CVJ for OCF has evolved from the use of sublaminar wires and cables [5,6] to modern-day screws, plates, and rod constructs, obviating the need for post-operative immobilization and early rehabilitation, and improving fusion rates. However, due to the limited bony surface, fusion around the CVJ is difficult, with significant rates of pseudoarthrosis and implant failures [7], especially in a certain high-risk group of patients with compromised anatomy and physiology, such as congenital bony anomalies, pathological fractures, and osteoporosis. 

The advantages of a multiple rod construct across the thoraco-lumbar and lumbo-sacral region for adult spine deformity, to enhance the stability, reduce rod stresses, and rates of implant failure have been described well in the literature [8-10]. But there is limited literature describing the same around the CVJ [11,12]. While our centre has achieved a high fusion rate (94.7%) [13], the analysis of the cases with incidences of pseudoarthrosis and implant failures reflected the need for a more rigid and stable construct. We hypothesize that a multiple rod construct for OCF may further enhance the stability and hence the fusion rate, especially in the aforementioned high-risk group of patients. Our experience with OCF [13] also suggests that new bony trabeculae formation is observed more frequently in the proximity of the connecting rods. Thus, we believe that a multiple rod construct would exhibit robust bony fusion.

We present our institutional experience with 10 patients who underwent OCF with a multiple-rod construct for various CVJ pathologies. With the help of enabling technologies such as an intra-operative navigation guidance system, we attempted to improve stability by increasing the number of anchors and placing additional rods. We retrospectively evaluated these patients for clinical and radiological outcomes, with a minimum follow-up of one year.

## Materials and methods

This is a retrospective evaluation of clinical and radiological outcomes of the patients who underwent OCF with a multiple rod construct, with a minimum follow-up of 12 months. The study protocol was approved by the Institutional Ethics Committee (SSHRI/CS/NS/MrodOCF/BRD/82/12.25, dated December 4, 2025) and was registered under Clinical Trials Registry - India (CTRI/2025/12/098899).

Data collection

Out of 2667 patients who underwent cervical spine surgery at Stavya Spine Hospital and Research Institute, Ahmedabad, India, we retrospectively evaluated the 10 patients who underwent OCF with a multiple rod construct, to date, with a minimum follow-up period of 12 months after surgery.

The primary inclusion criteria were patients with CVJ pathologies who underwent OCF with a multiple rod construct at our institute with a minimum follow-up period of 12 months. The patients who were lost to follow-up and those with inadequate follow-up records were excluded.

Demographic details and clinical data were documented from the hospital records. Radiological evaluation was performed based on lateral cervical spine radiographs, including the CVJ, and a CT scan at final follow-up.

Visual Analog Scale (VAS) [14] and Neck Disability Index (NDI) [15] were used for objective assessment of pain and disability, while the modified Japanese Orthopaedic Association score (mJOA) [16] and Nurick grading [17] were used to document disability due to myelopathy. Clinical assessment at presentation and at final follow-up was documented. All of the above-mentioned assessment tools are open access.

Radiological evaluation was done at presentation and at follow-up based on the lateral cervical spine radiographs. Occiput to axis (O-C2) lordosis, subaxial cervical spine (C2-C7) lordosis, and clivus-canal angle (CCA) were calculated. All the measurements were done by a single surgeon to avoid interobserver variability. CT scan of the CVJ at the final follow-up was evaluated to assess the bony fusion represented by the presence of the bony trabeculae between the occiput and the C2 lamina.

Data analysis

All the demographic, clinical, and radiological data were recorded in tabular form and analysed in Microsoft Excel (Microsoft Corporation, Redmond, WA, USA). Demographic and quantitative variables were summarized as mean ± standard deviation and range (minimum-maximum).

Surgical technique

After taking the baseline MEPs (Motor Evoked Potentials), the patient is carefully turned into the prone position with the reverse Trendelenberg position. Again, baseline MEPs and SSEPs (Somatosensory Evoked Potentials) are recorded. Routine exposure is done from the occiput to the subaxial cervical spine. Foramen magnum decompression is done with an ultrasonic bone scalpel. The navigation frame is now attached with a clamp to the spinous processes. Intra-operative 3D CT scan taken. With the help of intra-operative navigation guidance, pedicle screws are placed along the best possible trajectory, securing maximum screw purchase and avoiding complications such as screw breaches. C2 laminar screws are placed under navigation guidance to increase the number of anchors. Bilaterally, occipital plate-rods are connected to the cervical pedicle screws. Neck extension is done to reduce the atlanto-axial dislocation. Occipital plate-rods are fixed to the occiput. Distraction done at CVJ. Secondary rods are placed, connecting the C2 laminar screw to a polyaxial screw placed in the occiput, either in the midline or through one of the holes in the occipital plate-rod. Decortication of the C2 laminae is done. Autologous bone graft harvested from the iliac crest and placed over the CVJ.

## Results

The mean age of all the patients was 42.5±15.0 years, ranging from 23 to 67 years. There were seven female and three male patients included in the study. Eight patients underwent OCF for basilar invagination (BI) with atlanto-axial dislocation (AAD), while two patients had odontoid fracture along with atlas (C1) assimilation. The mean follow-up period was 14.5±5.97 months. Demographic data is represented in Table 1.

**Table 1:** Demographic data.

Variable	Category/measure	Value
Gender (n (%))	Male	7 (70%)
	Female	3 (30%)
	Total	10
Age (years)	Minimum	23
	Maximum	64
	Mean ± SD	42.5±15.0

All patients had clinical improvement, which was evident in all the clinical parameters assessed. Mean pre-operative VAS, NDI score, mJOA, and Nurick grades were 4.75±0.96, 23.5±4.20, 12.25±1.63, and 3.25±0.25, respectively, which improved to the mean values of 3.0±0.82, 14.75±0.65, 16.5±1.29, and 1.75±0.5, respectively, at the time of final follow-up, as shown in Table 2.

**Table 2:** Summary of clinical parameters at pre-operative time and at follow-up (n=10). Values are represented as mean±standard deviation. VAS = Visual Analog Scale, NDI = Neck Disability Index, mJOA score = Modified Japanese Orthopaedics Association score

Parameter	Value	
	Pre-op	Follow-up
VAS	4.75±0.96	3.0±0.82
NDI	23.5±4.20	14.75±0.65
mJOA score	12.25±1.63	16.5±1.29
Nurick grade	3.25±0.25	1.75±0.5

On evaluation of the radiological parameters, the mean values of pre-operative O-C2 lordosis in flexion and extension were 19.63°±6.34° and 28.08°±6.62°, respectively, and C2-C7 lordosis in flexion and extension were -5.95°±16.02° and 41.75°±8.94°, respectively, and CCA was 121.68°±7.16°. At follow-up, the mean O-C2 lordosis was found to be 17.35°±6.78°, and the mean C2-C7 lordosis was 17.23°±13.94°, while the mean CCA was 132.58°±6.50°. Radiological evaluation at pre-operative time and at follow-up is represented in Tables 3, 4.

**Table 3:** Summary of radiological parameters evaluated pre-operatively. Values are represented as mean±standard deviation. O-C2 lordosis = Occiput to C2 lordosis (lordosis at craniovertebral junction), C2-C7 lordosis = Subaxial cervical spine lordosis, CCA = Clivus-canal angle

Radiological parameter	Value (in degrees)
O-C2 lordosis (Flexion)	19.63±6.34
C2-C7 lordosis (Flexion)	-5.95±16.02
O-C2 lordosis (Extension)	28.08±6.62
C2-C7 lordosis (Extension)	41.75±8.94
CCA	121.68±7.16

**Table 4:** Summary of radiological parameters evaluated after minimum follow-up of 12 months. Values are represented as mean±standard deviation. O-C2 lordosis = Occiput to C2 lordosis (lordosis at craniovertebral junction), C2-C7 lordosis = Subaxial cervical spine lordosis, CCA = Clivus-canal angle

Radiological parameter	Value (in degrees)
O-C2 lordosis	17.35±6.78
C2-C7 lordosis	17.23±13.94
CCA	132.58±6.50

The status of fusion was assessed on a CT scan at follow-up. The continuous bony trabeculae between the occiput and the C2 laminae are suggestive of fusion. All ten patients exhibited bony trabeculae formation, indicative of fusion. No incidence of implant failure was noted at the early follow-up. None of the patients had any wound-related complications or infections.

Table 5 briefly summarizes the details of all the patients included in the study.

**Table 5:** Summary of all the cases. BI = Basilar invagination, AAD = Atlanto-axial dislocation, AAI = Atlanto-axial instability, ACM = Arnold-Chiari malformation

S. No.	Age	Gender	Diagnosis	Associated diagnosis	Procedure	Number of rods	Position of the addition rod
1	44	Female	BI with AAD	C1 assimilation, Fused C2-C3	O-C5 fusion	3	Midline occiput screw to left C2 lamina screw
2	36	Female	AAI	C1 assimilation	O-C4 fusion	3	Right occipital plate to left C2 lamina screw
3	40	Male	BI with AAD	Fused C2-C3	O-C5 fusion	3	Right occipital plate to left C2 lamina screw
4	50	Female	Pathological odontoid fracture	Multiple metastases from Ca Breast	O-C4 fusion	3	Midline occiput screw to right C2 lamina screw
5	38	Female	BI with AAI	C1 assimilation, Fused C2-C3	O-C5 fusion	3	Midline occiput screw to left C2 lamina screw
6	22	Female	Odontoid fracture non-union		O-C4 fusion	3	Midline occiput screw to right C2 lamina screw
7	63	Male	BI with AAD	C1 assimilation, Fused C2-C3	O-C3 fusion	3	Midline occiput screw to right C2 lamina screw
8	32	Male	BI with AAD	ACM	O-C5 fusion	3	Left occipital plate to right C2 lamina screw
9	66	Female	BI with AAD	C1 assimilation, Fused C2-C3	O-C4 fusion	4	Right occipital plate to left C2 lamina screw and left occipital plate to right C2 lamina screw
10	22	Male	BI with AAD	C1 assimilation, Fused C2-C3	O-C5 fusion	3	Midline occipital screw to right C2 lamina screw

## Discussion

**Figure 1:**
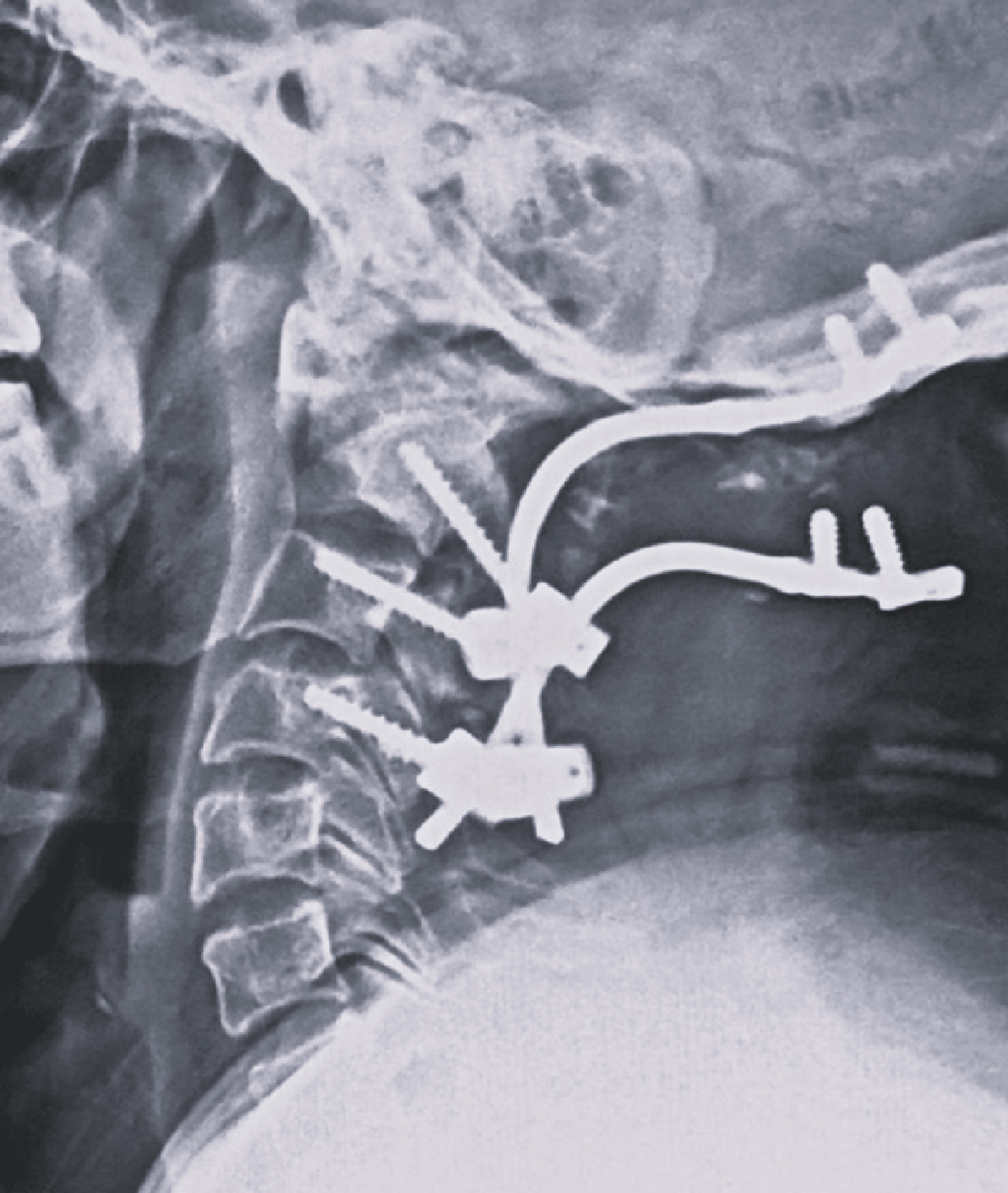
Lateral radiograph representing implant failure in a patient following OC fusion surgery for pathological odontoid fracture. OC fusion = Occipito-cervical fusion

Occipito-cervical fusion has evolved over many years from the uninstrumented fusion with strut autograft [4], to the use of wires and cables [5,6], and then to the modern-day screws, plate, and rod construct [18-20], providing rigid internal fixation and enabling early mobilization. However, this region is inherently difficult to achieve fusion, owing to the limited surface area available for bony fusion to occur, in addition to the associated complex anatomy and biomechanics. In a systematic review, Winegar et al. reported the rate of implant failure and pseudoarthrosis around 7-8% [2]. The compromised anatomy and physiology of the patients, such as rheumatoid arthritis or pathological fracture due to metastasis, further increases the risk of pseudoarthrosis [12] (Figure 1). 

Although the advantages of a multiple rod construct have been described widely in the literature [8-10] concerning the adult spinal deformity and osteotomies, the application of the same across the occipito-cranial junction is still limited. This could be attributed to the limited anchors that are available to place an additional rod. Also, extensive lateral dissection would be required to place a secondary rod laterally. We avoided the excessive lateral dissection by placing the additional secondary rod(s) medial to the primary rods, with the C2 laminae screws, which can be placed quickly and safely under intra-operative navigation guidance. 

With the use of enabling technologies such as intra-operative CT scan and navigation guidance, multiple anchor points in the axis (C2) and sub-axial cervical spine can be exploited for screw placement. Intra-operative navigation guidance enables the surgeon to place the C2 pedicle or pars screw, along with the lamina screw, together at once with precision and safety. While some of these anchors are connected to the occipital plate/rod on either side, others can be utilized to place a separate rod connecting to the occiput, creating a multiple rod construct. Additionally, many of these patients have certain congenital anomalies that may be missed on pre-operative CT scans (e.g., pedicle hypoplasia and high-riding vertebral artery). Intra-operative 3D CT scan enables us to detect these anomalies during the surgery and then modify the surgical plan, making the procedure safer and more efficacious. For example, if the C2 pedicle appears to be non-cannulable, either the C2 pars or lamina screw can be placed, or the C3 pedicle may be instrumented. 

**Figure 2:**
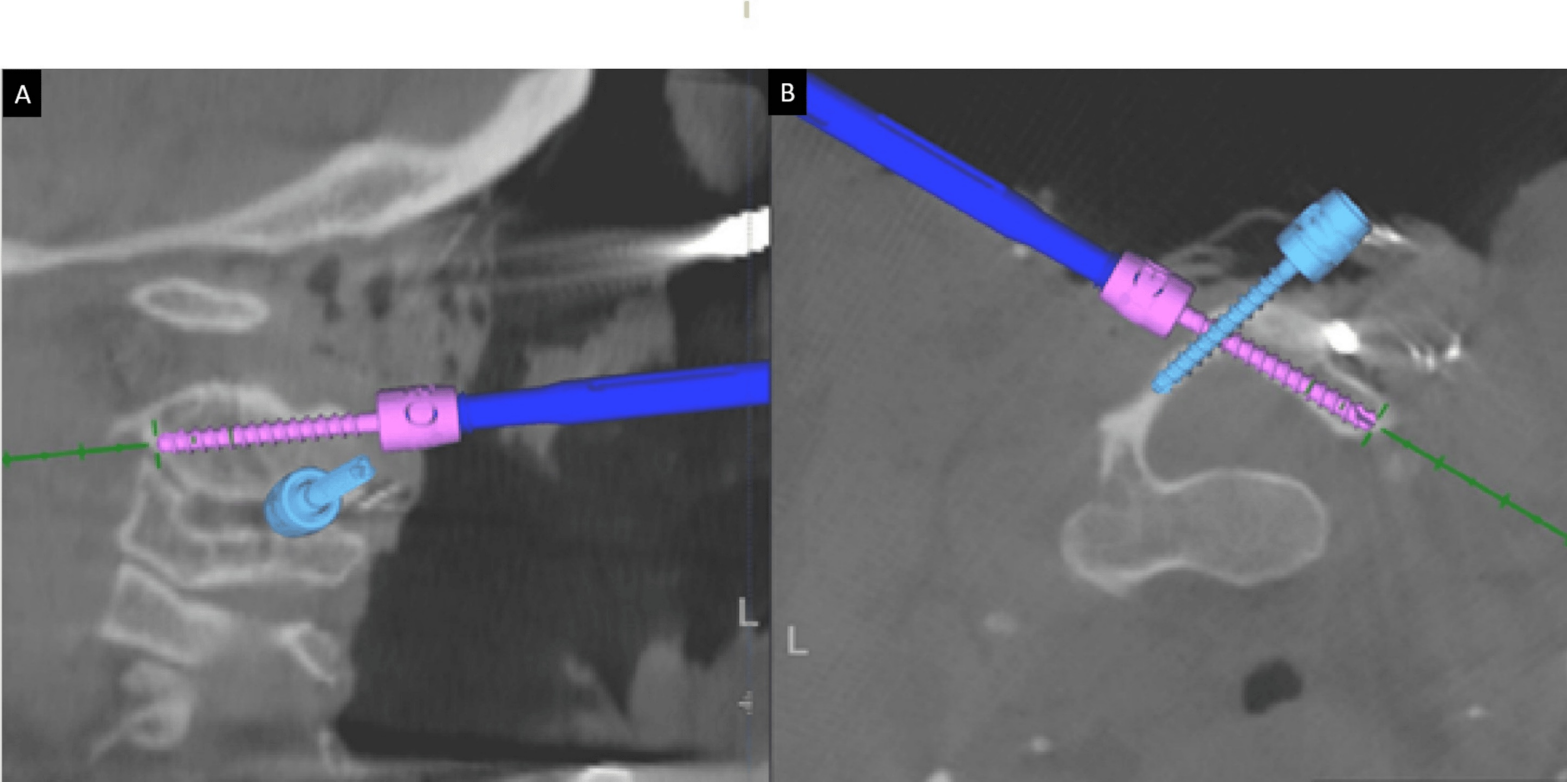
Demonstration of the placement of bilateral C2 laminar screws with the help of intra-operative navigation guidance. After placing the screw on one side, its trajectory can be saved for use as a reference to place the other side screw, without any hindrance. (A) shows the trajectories of bilateral C2 laminar screws in the oblique sagittal plane, whereas (B) represents the same in the axial plane.

Our experience with enabling technologies and IOTSS (Integrated Operation Theater and Spine Suite) has enabled us to perform complex surgeries in a safer and more efficacious way in quick time. Ultrasonic bone scalpel is used routinely for foramen magnum decompression and cervical laminectomy, if needed, to take precise bone cuts rapidly and safely, while avoiding bleeding from the cut bone margins. Intra-operative navigation guidance has enabled us to capitalise on more anchor points (Figure 2) and to place the screws in the best possible trajectory to secure strong anchor points, and ultimately a rigid internal fixation. C2 provides multiple possible anchor points, such as the C2 pedicle, the pars, and the laminae, as described above. Liu et al. described a case report where they described the placement of the C2 spinous process screw to get an additional anchor point to connect the third rod. However, the authors acknowledge the risk of spinal canal violation [21].

Collins et al. undertook the only described biomechanical study of a multiple rod construct for occipito-cervical fusion in the literature, comparing four constructs [11]. They used the occipital plate with dual-head occipital tulips and an offset connector to place a secondary rod(s). They reported that the multiple rod construct reduces the stresses on the primary rods, as well as on the occipital and subaxial cervical spine screws. They also reported a good outcome with no incidence of implant failure in their case series of 10 patients, who were followed up for a minimum period of one year.

Another description in literature is from Eto et al., where they describe a novel technique for a multiple rod construct across the occipito-cervical junction, and report two cases of failure of primary surgery within six months, who eventually underwent revision with a multiple rod construct as described [12]. They used an occipital plate with three holes in the midline. A 4.5 mm polyaxial screw is placed in one of these holes, while cortical screws are placed in the other two. An offset connector is used to connect the secondary rod between the polyaxial occipital screw tulip and the primary rod.

**Figure 3:**
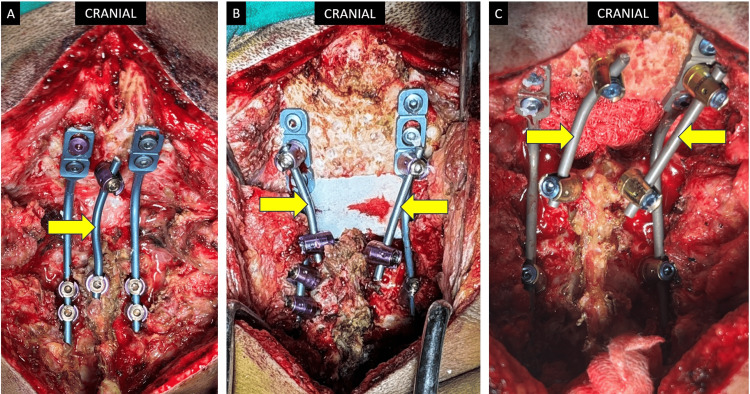
Intra-operative photos with various methods for multiple-rod construct for OC fusion. (A) A three-rod construct with the yellow arrow pointing towards the secondary rod placed between the midline occipital screw and the right C2 laminar screw. (B) A four-rod construct with the yellow arrows pointing towards secondary rods placed between polyaxial screws placed through occipital plates and C2 laminar screws on either side. (C) A four-rod construct is made with the yellow arrows pointing towards secondary rods. The midline occipital screw is connected to the right C2 laminar screw, while another rod is placed between the polyaxial screw placed through the right occipital plate and the left C2 laminar screw. OC fusion = Occipito-cervical fusion

In our experience, we have used multiple methods to secure an additional rod in the occipito-cervical fixation construct with the paramedian occipital plate/rods and screws (Figure 3), as follows: (1) Placing a 3.5 mm polyaxial screw in the midline of the occiput and connecting it with the C2 laminar screw; and (2) placing a polyaxial screw through one of the holes in the occipital plate/rod, and connecting it with the C2 laminar screw.

**Figure 4:**
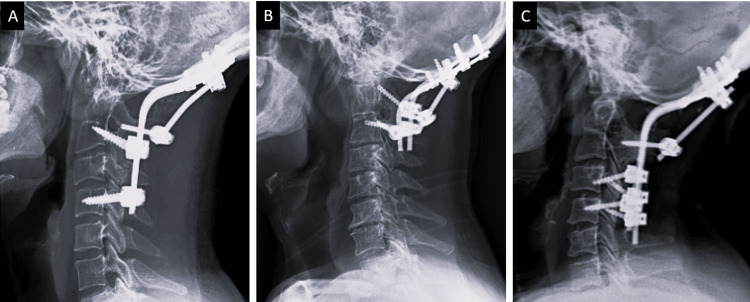
Representative follow-up lateral radiographs of patients operated for OC fusion with multiple rod construct. (A) Lateral radiograph of cervical spine at 12 months follow-up, who underwent occiput-C5 fusion with a three-rod construct. (B) Another patient's lateral radiograph of cervical spine at 14 months follow-up who underwent O-C3 fusion. (C) Seventeen months follow-up lateral radiograph of another patient operated on with O-C5 fusion. All the patients exhibit fusion and improvement in radiological parameters. OC fusion = Occipito-cervical fusion

All of the patients in our study showed clinical as well as radiological improvements (Figure 4) in follow-up. None of the patients showed incidences of implant failure or pseudoarthrosis in the early follow-up period. The follow-up CT scan has shown fusion in all patients, as evidenced by trabecular bone formation. These are consistent with the results of existing case series [11,12,21]. Although Liu et al. [21] mentioned their concern regarding the reduced surface area available for fusion with an additional midline rod, and the interference of the implant with the formation and maturation of the fusion mass [22], the outcomes of Eto et al. [12] did not suggest any such interference.

This study has several limitations. The study design is retrospective, without a control group for comparison, the sample size is small, and the follow-up period is short, with heterogeneity in multiple rod construct designs among patients (depending on the individual patient's anatomy and number of anchors available). The study is done at a single centre, by a single surgeon, not consecutively, which may lead to selection ambiguity. Without a control group, we cannot conclude that the multi-rod construct is superior to standard constructs. The heterogeneity of techniques and potential for observer bias further limit interpretation. These factors may limit the generalization of the results. However, a thorough evaluation of clinical and radiological outcomes is the strength of the study.

The purpose of this study is to share our relatively early experience with the multiple rod construct for occipito-cervical fusion, which we believe has evolved from the use of enabling technologies like intra-operative navigation guidance over the years. Further biomechanical and clinical, prospective, multi-centric studies with a larger sample size and longer follow-up period are recommended to reiterate the advantages of this new technique.

## Conclusions

Our early experience with multiple rod constructs for stable internal fixation for occipito-cervical fusion has shown clinical and radiological improvement without any incidences of implant failure in the early follow-up period. We believe that such a complex procedure is well executed in a safe and efficacious manner, with the help of IOTSS, with integrated enabling technologies such as intra-operative neuromonitoring, the ultrasonic bone scalpel, the intra-operative 3D CT, and navigation system. The limited literature has demonstrated biomechanical advantages, and our experience is consistent with the outcomes reported in case reports and case series, with no incidence of implant failure.
